# Comparing single oral contrast-enhanced ultrasonography and double contrast-enhanced ultrasonography in the preoperative Borrmann classification of advanced gastric cancer

**DOI:** 10.18632/oncotarget.23819

**Published:** 2017-12-22

**Authors:** Liang Wang, XiaoHua Wang, HongJu Kou, HuiLiao He, Mingdong Lu, LingLing Zhou, Yan Yang

**Affiliations:** ^1^ Department of Ultrasound, The Second Affiliated Hospital and Yuying Children’ s Hospital of Wenzhou Medical University, Wenzhou 325027, China; ^2^ Department of Gastrointestinal Surgery, The Second Affiliated Hospital and Yuying Children’s Hospital of Wenzhou Medical University, Wenzhou 325027, China; ^3^ Department of Pathology, The Second Affiliated Hospital and Yuying Children’s Hospital of Wenzhou Medical University, Wenzhou 325027, China

**Keywords:** ultrasonography, examination, advanced gastric cancer, Borrmann classification, surgery

## Abstract

**Objectives:**

To evaluate the accuracy of double contrast-enhanced ultrasonography (DCEUS) in preoperative Borrmann classification of advanced gastric cancer (AGC).

**Materials and Methods:**

A total of 162 patients histologically confirmed AGC were enrolled into this study. Single oral contrast-enhanced ultrasonography (SOCEUS) were performed in 80 patients and DCEUS (intravenous microbubbles combined with oral contrast-enhanced ultrasound) were performed in 82 patients preoperatively. The findings of the histopathologic examination of resected specimens after surgery were considered as gold standard. The accuracy of SOCEUS was compared with the accuracy of DCEUS in determining Borrmann classification. Interobserver agreement between two sonographyers of SOCEUS and DCEUS had also been assessed.

**Results:**

The accuracy of SOCEUS and DCEUS in Borrmann classification of advanced gastric cancer were 78.75% and 91.46% respectively. There was a significant difference between two methods (χ^2^ = 5.186, *P* < 0.05). The interobserver agreement of two methods was both excellent for assessing the Borrmann classification with a Kappa value of 0.777 by SOCEUS and 0.844 by DCEUS.

**Conclusions:**

DCEUS is a valuable method for Borrmann classification with its high accuracy preoperatively. It should be used widely.

## INTRODUCTION

Gastric cancer affects almost 1,000,000 individuals per year and remains the third most frequent cause of cancer deaths worldwide [1, 2]. Generally, east Asian countries (including Japan, Korea, and China) have high incidence of gastric cancer (i.e. > 40 cases per 100 000 men) [3, 4]. Recently, the outcome of gastric carcinoma post-treatment has been markedly improved with the development of diagnostic imaging modalities, increased early detection, and popularization of standard surgical methods. Nevertheless, advanced gastric carcinoma remains a disease with a poor prognosis [5]. Surgical resection is the most suitable treatment for the disease [6]. There should be reasonable and individualized comprehensive treatment strategies for patients with gastric cancer [7]. To classify the advanced gastric cancer is one of the crucial factors of therapeutic strategy. The classification of advanced gastric cancer according to Borrmann’s criteria is still widely used around the world [8]. Accurate Borrmann classification and cost-efficient preoperative evaluation have been required for the development of a reasonable operation program and assessment of the prognosis.

There are many modalities, such as computed tomography (CT), magnetic resonance imaging (MRI) and endoscopy, that have been used for assessing the Borrmann classification of AGC. But each of above modalities has its disadvantages: CT examination carries a burden on ionizing radiation; The cost of MRI examination is relatively high, and MRI examination has some contraindications, such as pacemakers or cochlea implants; In patients with Borrmann IV gastric cancer, the tumors grow typically in the submucosal, it is difficult for endoscopists to recognize the lesions in endoscopic examination.

Therefore, it is required to build up a non-invasive, well-reproducible, precise diagnostic procedure without radiation in modern times. SonoVue is a second generation contrast agent. It is an injection of sulfur hexafluoride microbubbles [9]. Combining ultrasonic oral contrast agent(UOCA) and intravenous microbubbles to check patients, we can easily find gastric malignancy and give a precise Borrmann classification. This paper summarized 162 cases of AGC and compared the findings of DCEUS and SOCEUS with surgical results to explore the value of DCEUS in Borrmann classification.

## RESULTS

Gastrectomy was performed in all 162 patients. The diameters of the resected gastric tumors ranged 1.1-13.5cm (mean 5.6±1.8cm). The histological classifications were well-differentiated adenocarcinoma (19 cases), moderately differentiated adenocarcinoma (36 cases), poorly differentiated adenocarcinoma (65 cases), undifferentiated carcinoma (12 cases), signet-ring cell carcinoma (28 cases), squamous carcinoma (2 cases). Among them, the pathological Borrmann classification in 20 cases (12.34%) was type I; in 48 cases (29.63%) was type II; in 71 cases (43.83%) was type III, and in 23 cases (14.20%) was type IV. For SOCEUS group, Borrmann classification in 9 cases (11.25%) was type I; in 22 cases (27.50%) was type II; in 38 cases (47.50%) was type III, and in 11 cases (13.75%) was type IV (Table 1). For DCEUS group, Borrmann classification in 11 cases (13.41%) was type I; in 26 cases (31.71%) was type II; in 33 cases (40.24%) was type III, and in 12 cases (14.63%) was type IV (Table 2). A lesion-by-lesion analysis revealed that 78.75% (63 of 80) gastric cancers were correctly classified by SOCEUS (Table 1) and 91.46% (75 of 82) gastric cancers were correctly classified by DCEUS (Table 2) respectively.

**Table 1:** The findings of SOCEUS compared with postoperative pathological results

SOCEUS	Postoperative pathological results			
Classification	Borrmann I	Borrmann II	Borrmann III	Borrmann IV
Borrmann I	8	1	1	0
Borrmann II	1	17	6	0
Borrmann III	0	4	29	2
Borrmann IV	0	0	2	9
Total	9	22	38	11
Accuracy	88.89%	77.27%	76.32%	81.82%

SOCEUS single oral contrast-enhanced ultrasonography. The overall accuracy of SOCEUS in Borrmann classification was 78.75% (63 of 80 patients).

**Table 2:** The findings of DCEUS compared with postoperative pathological results

DCEUS	Postoperative pathological results				
Classification		Borrmann I	Borrmann II	Borrmann III	Borrmann IV
Borrmann I		10	0	0	0
Borrmann II		1	24	2	0
Borrmann III		0	2	30	1
Borrmann IV		0	0	1	11
Total		11	26	33	12
Accuracy		90.91%	92.31%	90.91%	91.67%

DCEUS double contrast-enhanced ultrasonography. The overall accuracy of DCEUS in Borrmann classification was 91.46% (75 of 82 patients).

The interobserver reproducibilities were both almost excellent for assessing the Borrmann classification of AGC with a Kappa value of 0.777 (*P* = 0.000, 95% CI: 0.653, 0.893) by SOCEUS (Table 3) and 0.844 (*P* = 0.000, 95% CI: 0.729, 0.933) by DCEUS (Table 4). When the accuracy of SOCEUS were compared with the accuracy of DCEUS, significant difference was found (Table 5).

**Table 3:** Concordance of Borrmann classification by SOCEUS according to the findings of two observers

Observer A					
Observer B	Borrmann I	Borrmann II	Borrmann III	Borrmann IV	Total
Borrmann I	6	2	1	0	9
Borrmann II	1	20	2	0	23
Borrmann III	1	2	32	2	37
Borrmann IV	0	0	1	10	11
Total	8	24	36	12	80

The inter-observer reproducibility was almost perfect for Borrmann classification of advanced gastric cancer by SOCEUS with a Kappa value of 0.777 (*P* = 0.000, 95% CI: 0.653, 0.893).

**Table 4:** Concordance of Borrmann classification by DCEUS according to the findings of two observers

Observer A					
Observer B	Borrmann I	Borrmann II	Borrmann III	Borrmann IV	Total
Borrmann I	10	1	0	0	11
Borrmann II	2	24	2	0	28
Borrmann III	0	2	28	1	31
Borrmann IV	0	0	1	11	2
Total	12	27	31	12	82

The inter-observer reproducibility was almost perfect for Borrmann classification of advanced gastric cancer by DCEUS with a Kappa value of 0.844 (*P* = 0.000, 95% CI: 0.729, 0.933).

**Table 5:** Comparison of the two methods in Borrmann classification

	Accurate	Inaccurate	Total	Accuracy
SOCEUS	63	17	80	78.75%
DCEUS	75	7	8291	46%
Total	138	24	16285	19%

χ^2^ = 5.186, *P* < 0.05.

## DISCUSSION

The classification of advanced gastric cancer by Borrmann in 1926 into 4 types is still accepted worldwide by endoscopists, radiologists and surgeons [8, 10]. The classification is very simple and straightforward in expressing the morphological characteristics of advanced gastric cancer. Furthermore, it has a certain link with the pathological types of cancer and has important clinical values in determining the biological behavior of gastric cancer, clinical prognosis and so on. In recent years, with the development of ultrasonic equipments and technology, ultrasonographers have accumulated many experiences in observing sub-type of gastric cancer with the application of real-time ultrasound imaging. But conventional ultrasound examination is easily affected by tissue movements and respiration of the patient, it is difficult to detect tumors with small size and deep location. It is reported that the use of water as a distending agent for the stomach, with bolus administration of intravenous contrast, improves the accuracy of CT for staging gastric cancer [11, 12]. This study used UOCA and SonoVue as contrast agents to check patients with gastric cancer and gave Borrmann classification of tumors preoperatively, which was much better than traditional transabdominal ultrasonography.

It is very important to pinpoint the location of the stomach and measure the thickness of the gastric wall when we detect the presence and extent of a variety of gastric disorders such as carcinoma [13]. The radiologic features of gastric malignancy include intraluminal masses, thickening of the gastric wall, prominent and irregular gastric rugae, and large ulcers with irregular margins and elevated borders [14]. When the stomach is filled with UOCA, the gas in stomach is discharged, a uniform distribution of good sound transmission interface is performed. That leads to ultrasonic artifacts decreasing and a clear gastric wall displaying, so as to increase the detection rate of the diseases of stomach and adjacent organs. A wall thickness > 1 cm is considered abnormal when the stomach is well distended [15]. Table 1 showed the overall accuracy of SOCEUS in determining the Borrmann classification of AGC was 78.75%, and the accuracy of type I, II, III, IV were 88.89%, 77.27%, 76.32%, 81.82% respectively. It is difficult for SOCEUS to distinguish tumor tissues from peritumoral inflammation and fibrosis because of the limitation of resolution and the small acoustic impedance difference, and this is the most common reason for misdiagnosis [16–20]. SOCEUS detects the tumors based on the shape changes of gastric wall and gastric cavity, so it’s sensitive to intracavity elevated masses and diffuse gastric wall thickening. However, SOCEUS couldn’t effectively demonstrate the micro-invasion of the tumors and histologic changes of layers of gastric wall. Thus, SOCEUS is more accurate in Borrmann type I and type IV, and less accurate in Borrmann type II and type III.

Angiogenesis and infiltration are the characteristics of the tumor invasive growth [21]. SOCEUS is unable to display the microvascular perfusion of the lesions. SonoVue is a suspension of phospholipid stabilized sulphur hexafluoride (SF6) microbubbles, which can enter the capillary vessels of gastric cancer through blood circulation [9, 22]. SonoVue produces strong echogenicity over the range of frequencies used in medical ultrasound examinations. Using UOCA and SonoVue dual ultrasonic agents is beneficial to classify the tumors preoperatively. The overall accuracy of DCEUS in determining the Borrmann classification of AGC was 91.46%, and the accuracy of type I, II, III, IV were 90.91%, 92.31%, 90.91%, 91.67% respectively. Lesions enhanced in the arterial phase after the injection of intravenous contrast agents. Agents washed out in the venous phase, which made the borders of malignancy more clear. Thus we could easily see the tumor’s shape and judge the depth of invasion. So DCEUS is more accurate than SOCEUS (χ2 = 5.186, *P* < 0.05). In addition, interobserver reproducibility of DCEUS was calculated and kappa value was 0.844 which represented excellent agreement.

Borrmann IV gastric cancer is characterized by a diffiuse thickening and sclerosis of the gastric wall, marked hypertrophy of the mucosal folds and erosions [23]. It usually has a poor prognosis, the 5-year survival rate after gastrectomy has been reported to be approximately 30% [24, 25]. The infiltrative carcinoma may grow either superficially over the surface of the mucosa or permeate the entire thickness of the wall, producing a characteristic tumour pattern known as linitis plastica [26]. In these patients, endoscopy has been reported to have a sensitivity of only 33%–73%. As the cancer cells grow typically in the submucosal layer in patients with linitis plastica, it is difficult for endoscopists to recognize the lesions. Therefore, the overlying mucosa appears normal or only slightly affected and cancer cells are often not present in mucosal biopsies for Borrmann IV cancer [27, 28]. The advantage of DCEUS is the high-contrast resolution between tumors and normal tissues, which makes it sensitive for lesion detection, characterization, and staging. Thus DCEUS performed better than endoscopy in diagnosis of Borrmann IV.

Of course, DCEUS also has overestimate and underestimate in Borrmann classification. Tumor growth is irregular, ultrasound images of DCEUS are in different 2-D views, therefore, the three-dimensional structures of the tumor can’t be displayed completely and deviation may exist sometimes. Other factors, including the resolution of equipments, tumorous necrotic tissues without enhancement, may also affect the accuracy of diagnosis. It is worth mentioning that the arterial phase is short but the information it provided is very important, and sometimes we need to review the clips one image by one image to get important diagnostic information. Furthermore, we should study all phases before we draw a conclusion.

### Limitations

Our study was retrospective and we only enrolled patients referred to our hospital for surgery. Intraobserver reliability was not evaluated. And we didn’t compare DCEUS to CT, MRI or endoscopy in preoperative Borrmann classification. We think we should do these studies in our future research.

## MATERIALS AND METHODS

The study was conducted in accordance with the Declaration of Helsinki and was approved by the Ethics Committee of our hospital. Informed consent was obtained from all patients prior to their examinations.

### Patients

Between September 2013 and December 2016, 195 patients were diagnosed with gastric cancer in the Second Affiliated Hospital and Yuying Children’s Hospital of Wenzhou Medical University. 96 patients were examined by SOCEUS and 99 patients were examined by DCEUS preoperatively. The inclusion criteria were as follows: ① adenocarcinoma of stomach proven by endoscopic biopsy; ② not treated with chemotherapy, radiotherapy or immunotherapy previously; ③ surgical resections were performed within 1 week after the SOCEUS and DCEUS examinations. The exclusion criteria were as follows: ① elderly patients with comorbidities for surgery(7 cases); ② unresectable lesions with widespread metastasis (11 cases); ③ early stage tumors on postoperative pathology (15 cases). The final study consisted of 162 patients (53 females, 109 males, mean age 61.6 ± 11.2 years (range 32–80)). SOCEUS group consisted of 80 patients while DCEUS group consisted of 82 patients.

### Equipments, agents

Ultrasonographic studies were performed with Acuson Sequoia 512 system (Siemens, Mountain View, CA, USA), equipped with a 4V1 transducer (frequency: 1.0–4.0 MHz) and the microbubble-specific contrast pulse sequencing (CPS) technology; The ultrasonic oral contrast agent Xinzhang^®^ (Huqingyutang, HangZhou, China) was

composed by a kind of soya derivative (48 grams per package); Intravenous contrast agent SonoVue (Bracco, Italy) was an injection of sulfur hexafluoride microbubbles.

### Examination and observation

All patients fasted for 8–12 hours and received 0.5mg atropine pro injectione intramusculari 30 minutes before examinations in order to inhibit gastric peristalsis during examinations.

SOCEUS examination: A baseline ultrasonography of the stomach was performed in the fundamental mode by using 4V1 probe to identify each tumor. We also checked other abdominal organs to determine if there are any metastatic lesions. Then the patients ingested 500ml of UOCA, and were examined in the supine, left lateral and right lateral position during full inspiration. The gastric lesions were observed, the size of tumors were measured, the shapes and echo features of tumors were described. The images were digitally recorded on tapes.

DCEUS examination: DCEUS was based on SOCEUS. The previous steps were the same as SOCEUS examination. Further steps were carried out after the administration of 2.4 ml of Sonovue, as a bolus via a 19-gauge peripheral intravenous cannula, followed by a 3∼5ml saline flush. We performed DCEUS using the contrast pulse sequencing (CPS) mode. The settings were as follows: transmit frequency, 1.5 MHz; acoustic power, −15 to −21 dB; frame rate, 17–20. A low mechanical index (< 0.2) was selected, in order to avoid microbubble disruption. The enhancement patterns of the gastric lesions were stored up to 5 min, including the arterial, venous, and late phases. The images were digitally recorded on tapes, including the baseline SOCEUS images and dynamic DCEUS images of the target lesions.

### Image analysis and Borrmann classification

**Figure 1:**
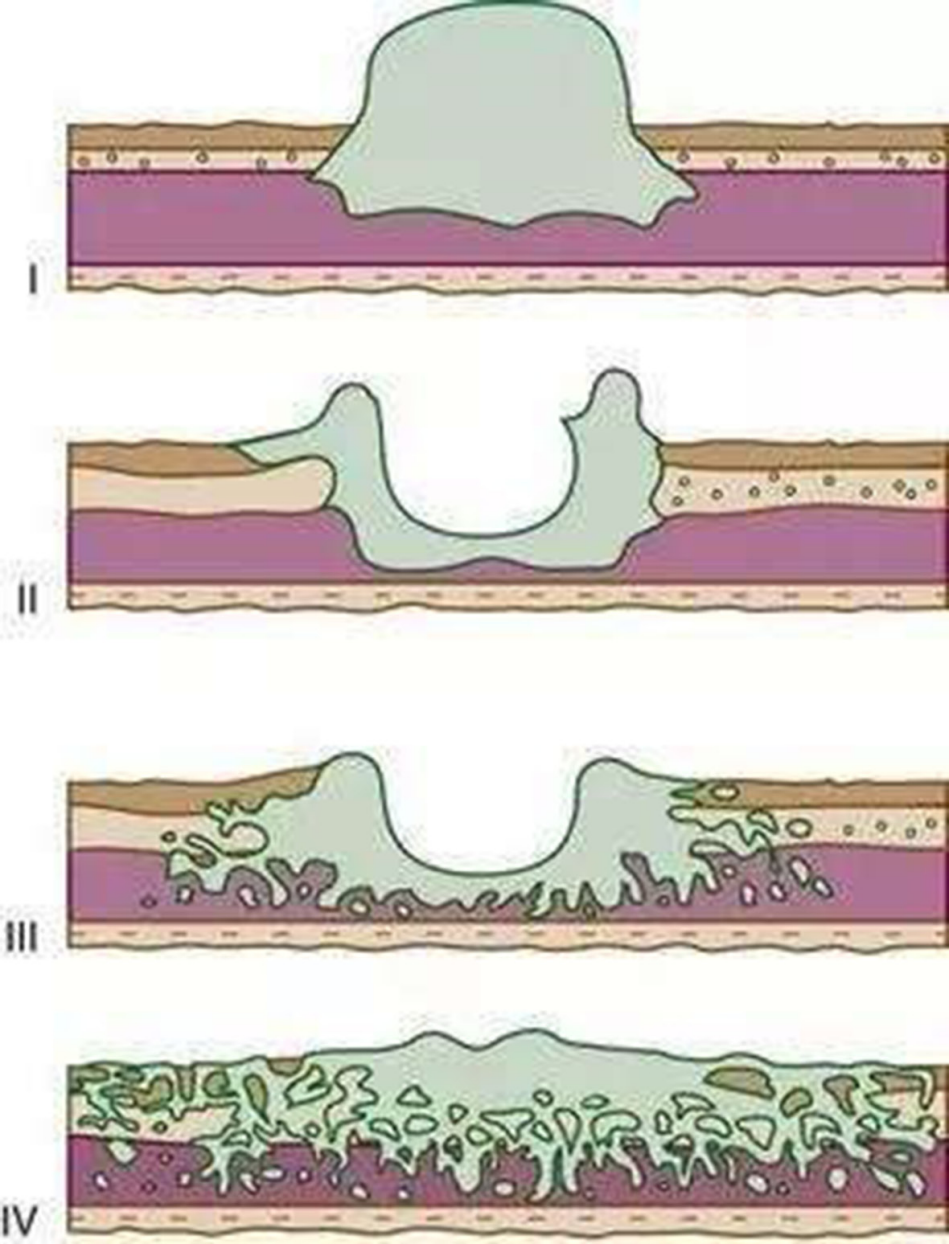
Diagrammatic sketch of Borrmann classification **I** polypoid or fungating type. **II** ulcerating lesions surrounded by elevated borders, **III** ulcerating lesions with invasion of the gastric wall, **IV** diffusely infiltrating (linitis plastica).

**Figure 2:**
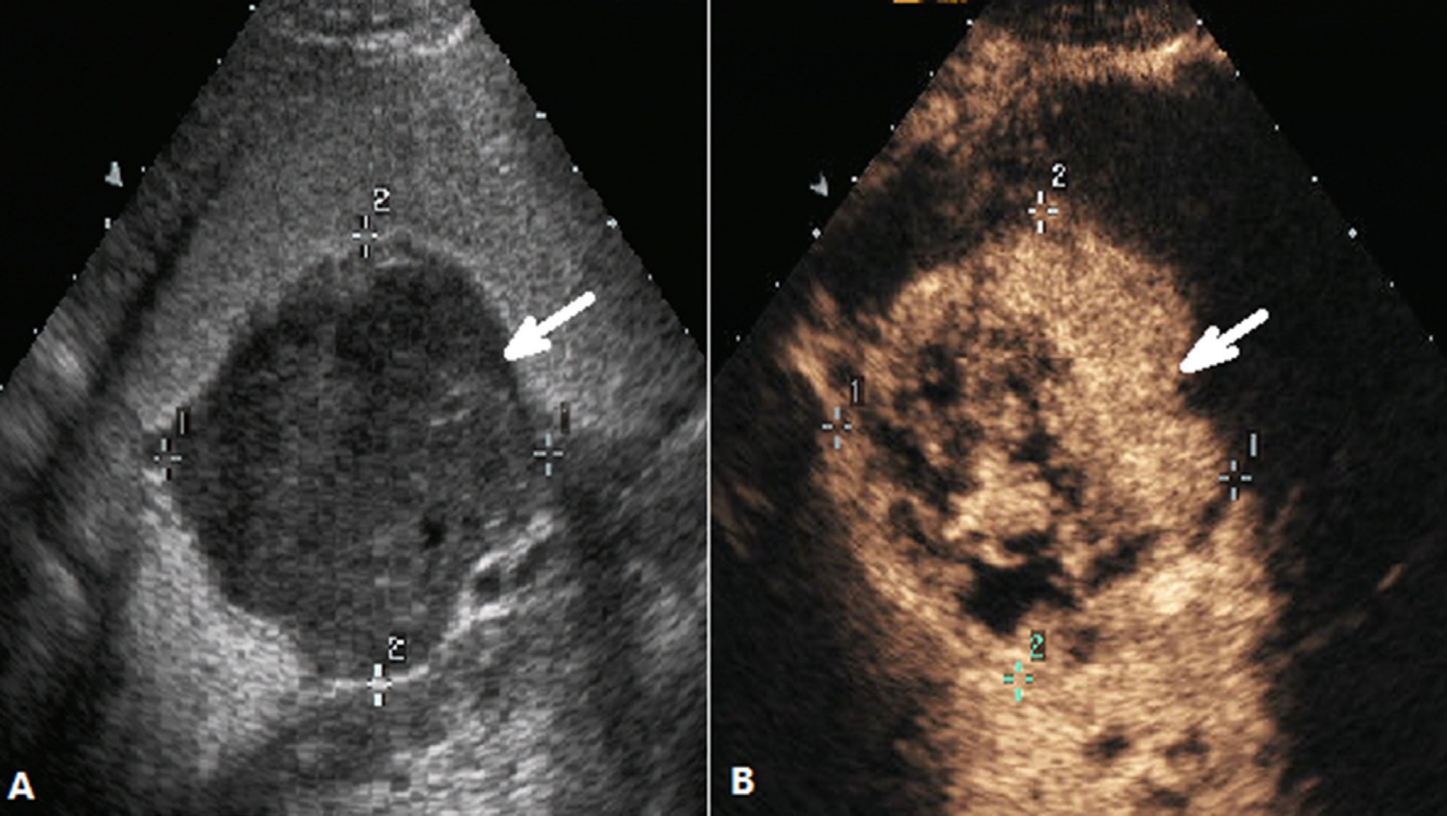
DCEUS images of a case classified as Borrmann type I (**A**) Ultrasonography showed the thickened gastric wall with nodular polypoid appearance(arrow) after the stamoch was filled with UOCA, (**B**) The same view showed that the lesion(arrow) was enhanced after administration of intravenous contrast agent.

**Figure 3:**
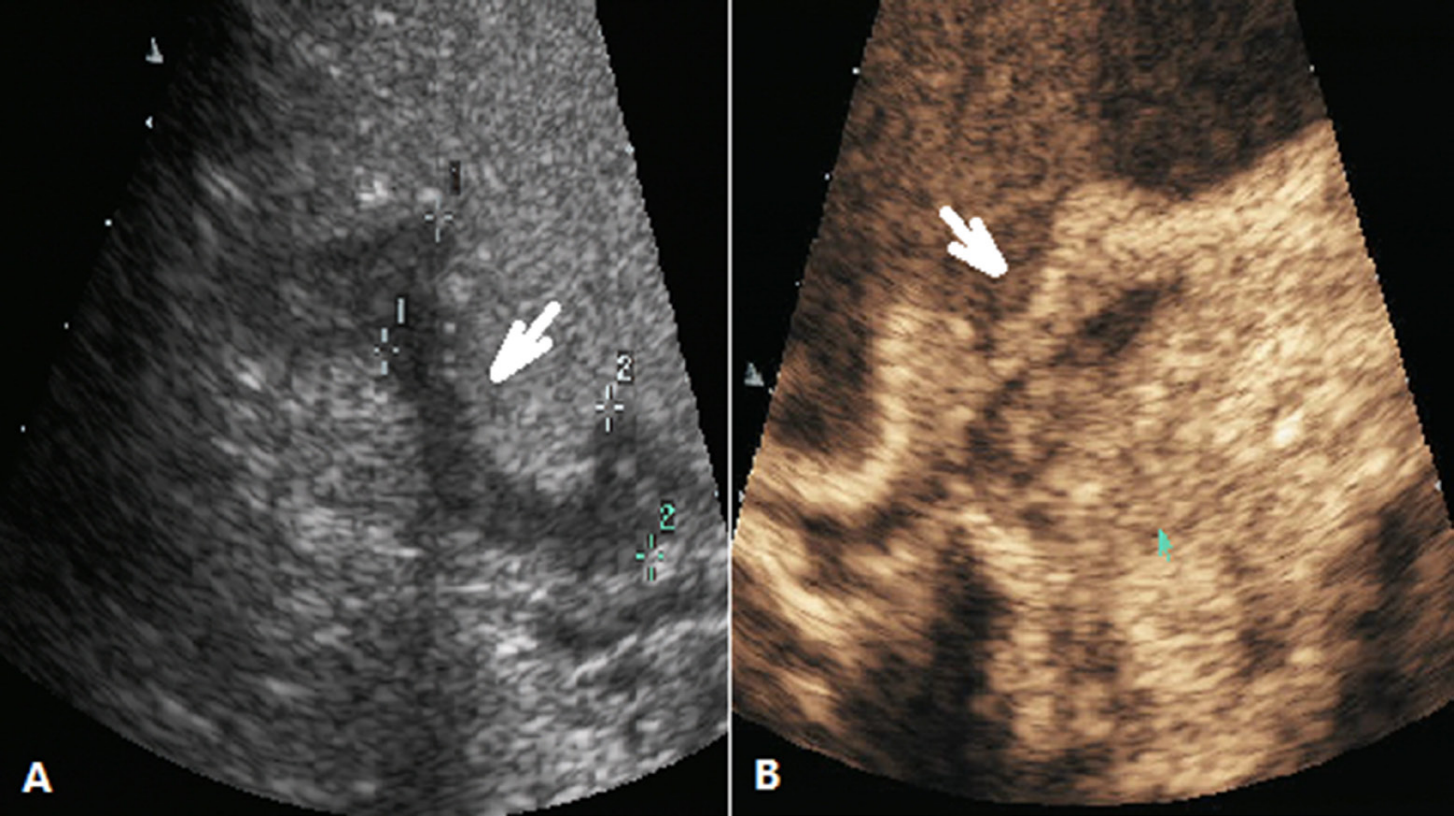
DCEUS images of a case classified as Borrmann type II (**A**) Ultrasonography showed the ulcerating lesion surrounded by elevated borders (arrow) after the stamoch was filled with UOCA, (**B**) showed the lesion(arrow) was enhanced after administration of Sonovue.

**Figure 4:**
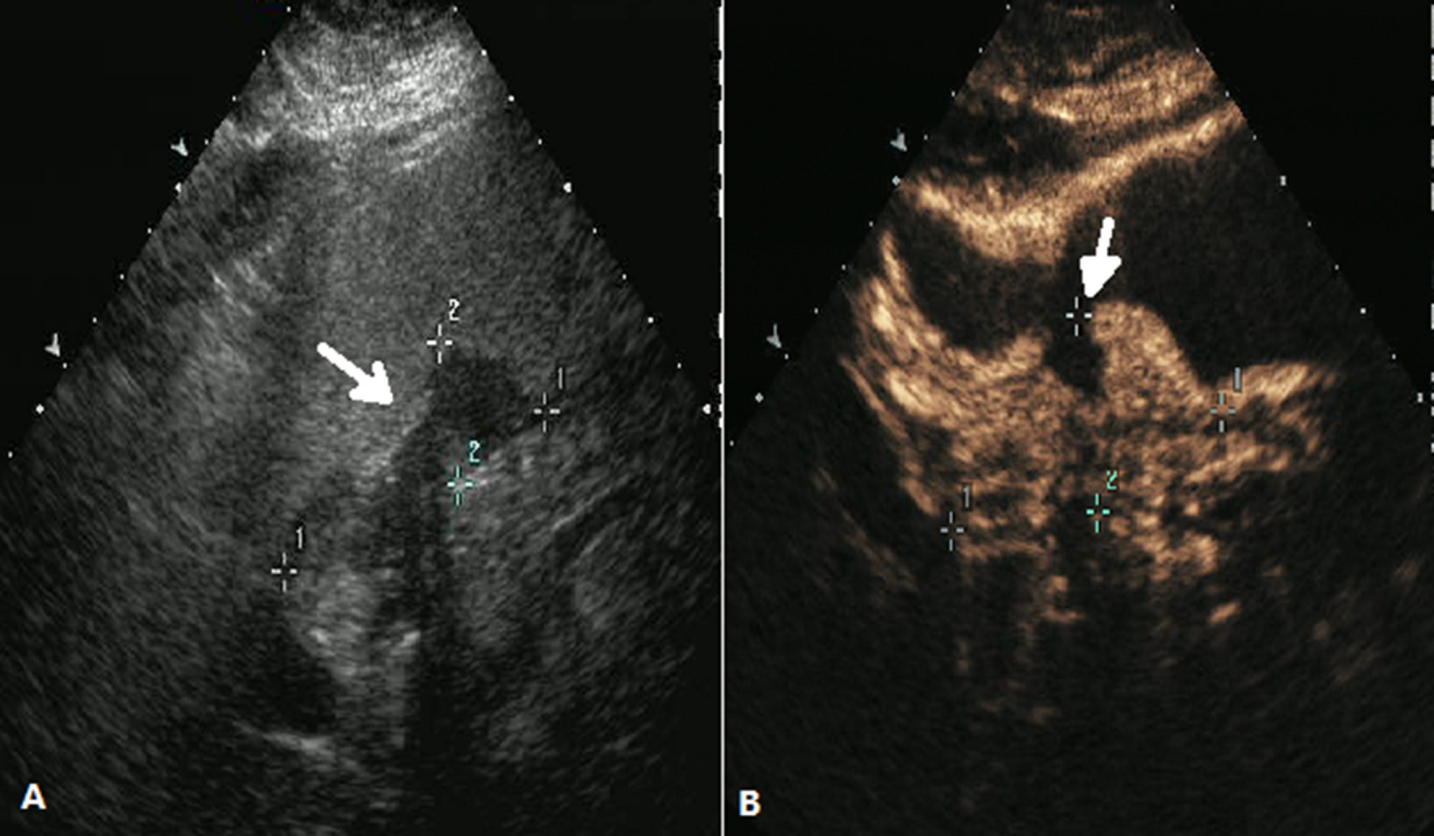
DCEUS images of a case classified as Borrmann type III (**A**) Ultrasonography showed the ulcerating lesion with invasion of the gastric wall (arrow) after the stamoch was filled with UOCA, (**B**) showed the lesion(arrow) was enhanced after administration of Sonovue.

**Figure 5:**
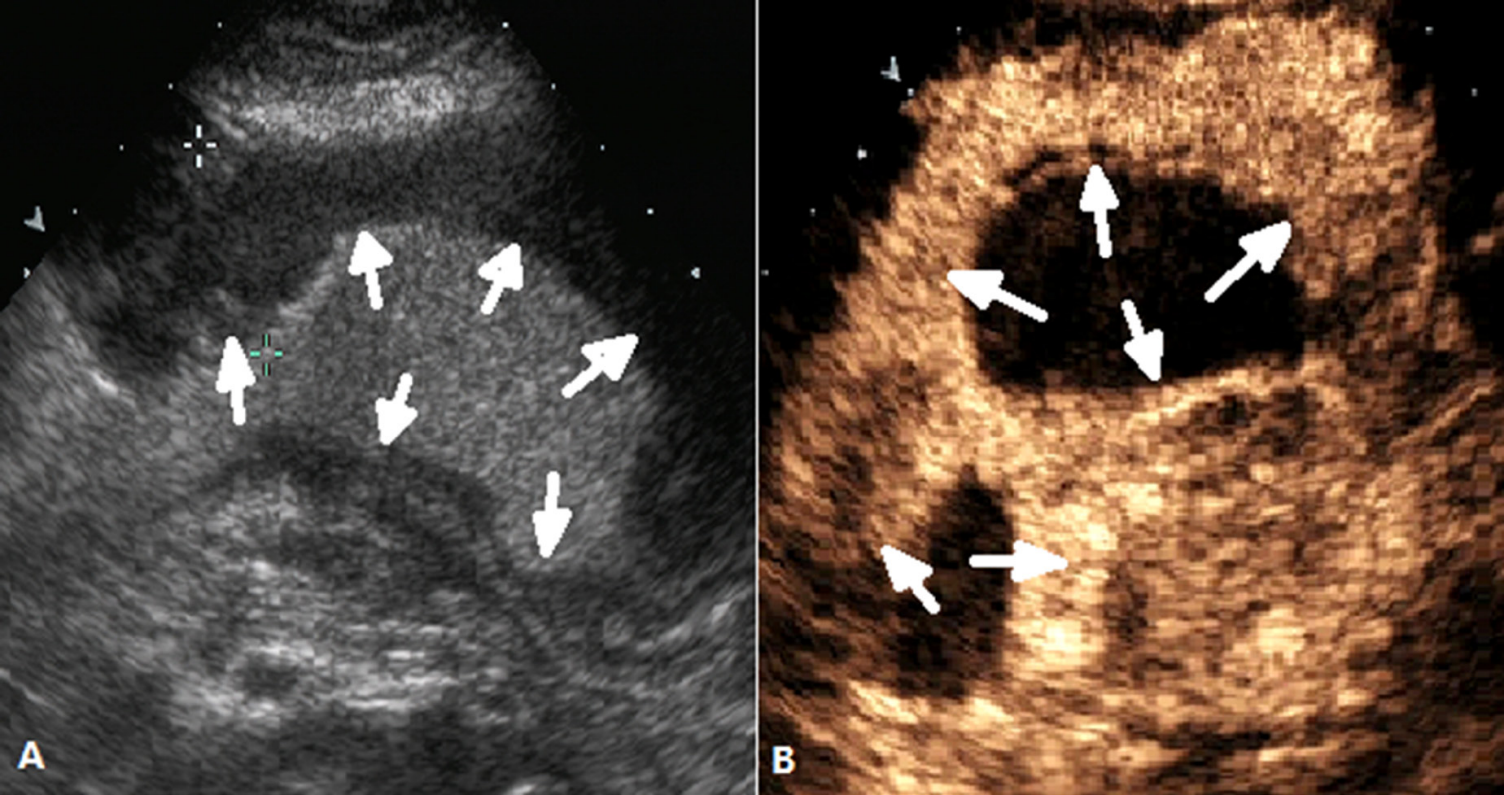
DCEUS images of a case classified as Borrmann type IV (**A**) and (**B**) showed a diffusely thickened gastric wall (arrow) .

These images were analyzed by two independent sonographers(X W and H H, X W analyzed the images of SOCEUS and H H analyzed the images of DCEUS). Both of them had more than 10 years of experience and were blinded to the patients’ clinical datas, other imaging findings, and pathology results at the time of the analysis. The tumor type was based on the Borrmann classification system (Figure 1) —classifying gastric carcinomas as polypoid or fungating (type I) (Figure 2), ulcerating (type II) (Figure 3), ulcerating-infiltrating (type III) (Figure 4) or infiltrating (type IV) (Figure 5). In patients with multiple lesions, only one lesion, the larger and more conspicuous one was considered for characterization, with a total of 162 gastric carcinoma evaluated.

For the interobsever agreement, the image datas of SOCEUS and DCEUS were analyzed again by another two sonographers (L W and Y Y, with 12 and 19 years of experience, respectively. L W analyzed the images of SOCEUS and Y Y analyzed the images of DCEUS). The results were compared with the previous findings(analyzed by X W and H H) for calculating the inter-observer agreement.

All patients underwent surgery. The findings of the histopathologic examination of resected specimens were considered as gold standard and were retrospectively compared with the results of SOCEUS and DCEUS.

### Statistical analysis

Statistical analyses were performed using SPSS version 22.0 (SPSS Inc., Chicago, IL, USA). The change of diagnostic performance from SOCEUS to DCEUS was assessed by chi-square test. Interobserver agreement between two blinded sonographyers of SOCEUS and DCEUS were assessed using Kappa analysis. A kappa value of 0.4 or more represented fair agreement, and a value of 0.75 or more represented excellent agreement. For all tests, *P* < 0.05 was considered to indicate a statistically significant difference.

## CONCLUSIONS

As a convenient and repeatable method, DCEUS has high accuracy in Borrmann classification of advanced gastric cancer. It could be a useful tool before surgery.

## Abbreviations

AGC: advanced gastric cancer
SOCEUS: single oral contrast-enhanced ultrasonography
DCEUS: double contrast-enhanced ultrasonography
UOCA: ultrasonic oral contrast agent
CT: computed tomography
MRI: magnetic resonance imaging
